# The role of the PKCζ/JNK signaling pathway in regulating the development of femoral head necrosis

**DOI:** 10.1590/1414-431X2025e13771

**Published:** 2025-03-03

**Authors:** Miaofeng Hu, Cheng Li, Qi Sun, Baisong Hu, Jiong Yang, Xiufeng Wang, Jinshan Huang, Di Shen

**Affiliations:** 1 Department of Orthopedics, Hangzhou Fuyang Hospital of Orthopedics of Traditional Chinese Medicine, Hangzhou, China; 2 Department of Orthopedics, Zhuji People's Hospital of Zhejiang Province, Shaoxing, China

**Keywords:** Osteonecrosis of the femoral head, Glucocorticoid, Osteoblast apoptosis, PKCζ, JNK

## Abstract

Osteonecrosis of the femoral head (ONFH) is a debilitating condition characterized by the death of bone cells in the hip joint, resulting in profound disability. This condition has a significant global prevalence. Glucocorticoid (GC)-induced apoptosis of bone cells serves as a crucial cellular mechanism underlying ONFH. The protein kinase C zeta (PKCζ) and c-Jun N-terminal kinase (JNK)/c-Jun cascades have been implicated in the progression of ONFH, yet their interrelationship and contributions to disease development remain unclear. The objective of this study was to investigate the combined impact of PKCζ and JNK/c-Jun signaling on dexamethasone (Dex)-induced apoptosis in osteoblasts *in vitro* and in GC-induced ONFH rat models *in vivo*. *In vitro* experiments were conducted using hFOB1.19 osteoblastic cells to scrutinize the effects of Dex-induced apoptosis. The role of the PKCζ/JNK/c-Jun signaling pathway in this process was examined using naringenin-7-O-β-D-Glucuronide (N7G), a PKC inhibitor, and anisomycin, a JNK activator. The findings were further validated using a rat model of ONFH *in vivo*. Our results revealed that PKCζ activation augmented JNK/c-Jun signaling and facilitated Dex-induced osteoblast apoptosis. Inhibition of PKCζ with N7G mitigated these effects, while JNK activation with anisomycin intensified them. Similar regulatory effects on osteoblast apoptosis and ONFH progression were observed in the *in vivo* rat models. Glucocorticoids can induce osteoblast apoptosis and contribute to the development of ONFH by activating the PKCζ/JNK/c-Jun signaling pathway. This study provides compelling evidence supporting the potential therapeutic value of comprehending the pathogenesis of ONFH and developing targeted treatments for this debilitating condition.

## Introduction

Osteonecrosis of the femoral head (ONFH) is a progressive condition that can result in significant pain and disability across all age groups (1). This ailment can stem from various factors such as alcohol abuse, smoking, trauma, and specific medical conditions. Among the prominent triggers for ONFH is the usage of dexamethasone (Dex) or other glucocorticoids (GC), commonly prescribed for treating inflammatory and autoimmune disorders like rheumatoid arthritis, lupus, and asthma. Research indicates that 9-40% of individuals undergoing long-term GC therapy experience osteonecrosis (1,2). The induction of bone cell apoptosis by GC stands out as a critical mechanism in the progression of ONFH, suggesting that inhibiting this process could present a viable approach for disease prevention or slowdown (3,4).

The disruption in the equilibrium between osteoblasts (bone-forming cells) and osteoclasts (bone-resorbing cells), fueled by osteoblast apoptosis, ultimately leads to ONFH (5,6). Recent evidence underscores the vulnerability of osteoblasts to GC-induced damage through apoptosis induction and malfunction during the onset of GC-induced ONFH. Consequently, unraveling the impact of GC on osteoblasts (3,4), as well as the involvement of PKCζ (7) and JNK/c-Jun (4) signaling pathways, holds promise for advancing ONFH treatment modalities.

In ONFH, the disruption of blood supply to the femoral head causes cellular hypoxia, resulting in the death of osteocytes and bone marrow cells (8,9). This hypoxia-induced cell death triggers the production of reactive oxygen species (ROS) such as superoxide, hydrogen peroxide, and hydroxyl radicals. Elevated ROS levels induce oxidative harm in cells - including lipid peroxidation, DNA damage, and protein denaturation - consequently triggering apoptosis. Investigations utilizing Dex-induced ONFH murine or cellular models have unveiled the involvement of ROS accumulation in osteoblast apoptosis (8-12). Administration of Dex amplifies ROS levels, with excessive ROS leading to osteoblast apoptosis via the Bax/Bcl2/caspase-3 apoptotic pathway.

Naringenin, abundant in citrus fruits like grapefruit, oranges, and lemons (13-15), is recognized for its antioxidant properties in combating oxidative reactions catalyzed by free radicals and transition metal ions. It may also exhibit anti-inflammatory effects by inhibiting specific enzymes such as cyclooxygenase. However, orally administered naringenin encounters limited bioavailability due to poor absorption and swift metabolism, while its metabolite naringenin-7-O-β-D-glucuronide (N7G) is better assimilated (14,15). Studies have indicated that naringenin can surmount radiotherapy resistance in cancer treatment *in vitro* by inhibiting ROS-induced protein kinase C-zeta (PKCζ) activation (13). PKCζ, a member of the atypical protein kinase C subfamily, plays crucial roles in cellular processes like cell growth, differentiation, and apoptosis (13,16-18). Recent research has demonstrated that reducing PKCζ expression shields osteoblasts from GC-induced ONFH (19). However, the anti-apoptotic efficacy of N7G in osteoblasts and ONFH remains underexplored. In this study, we aimed to evaluate the anti-apoptotic potential of N7G in Dex-induced rat osteoblasts, explore the involvement of PKCζ and JNK/c-Jun signaling pathways, and elucidate the underlying mechanisms.

## Material and Methods

### Cell line, chemicals, and reagents (*in vitro*)

The hFOB1.19 human osteoblasts were obtained from the Cell Bank of Shanghai Institute of Biological Science (China). All cell culture reagents were obtained from Gibco (China). Dex (200 μM, 24-h; Sigma Aldrich, China) and N7G (20-200 μM, 24-h; Sigma Aldrich) were used *in vitro*. All *in vitro* cell experiments were performed with three replicates.

### Establishment of the osteonecrosis model and treatment (*in vivo*)

Sprague-Dawley (SD) male rats (age: 8 weeks; weight: 220±20 g) were randomly and equally divided into the following four groups: 1) Control (untreated) group (n=10), 2) ONFH group (rats treated with Dex, n=10), 3) N7G group (ONFH rats treated with PKCζ inhibitor N7G (13), n=10), and 4) anisomycin group (ONFH rats treated with PKCζ inhibitor N7G and JNK agonist anisomycin (20), n=10). GC-induced ONFH models were established following a previously reported method (4). In summary, rats were intravenously injected with lipopolysaccharide (2 mg/kg per day, Sigma Aldrich) once daily for the initial two days, followed by intramuscular (gluteus muscles) injections of methylprednisolone (20 mg/kg/day, Pfizer, USA) once daily for three consecutive days. For the N7G and anisomycin groups, the PKCζ inhibitor N7G (Sigma Aldrich) was dissolved in warm water for oral administration at a dosage of 210 mg/kg twice daily, following a previously described method (13). For the anisomycin group, the JNK agonist anisomycin (Sigma Aldrich) was administered via subcutaneous injection at a dosage of 150 mg/kg per day, in accordance with previously established methods (13,20).

None of the rats died before the scheduled euthanasia, which was performed under 4% chloral hydrate anesthesia. Next, the femoral heads were collected for micro-computed tomography (CT) scanning, immunostaining, and immunohistochemical staining. All the experimental and animal care procedures were approved by the Animal Research Ethics Committee of Hangzhou Fuyang Hospital of Orthopedics of Traditional Chinese Medicine (No. 2022-0039) and were in compliance with the National Institutes of Health Guidelines for the Care and Use of Laboratory Animals (USA).

### PKC**ζ** knockdown (*in vitro*)

For knockdown of PKCζ, the shRNA sequence targeting PKCζ (shPKCζ, 5′-GGCCATGAGCATCTCTGTTGT-3′) was subcloned into the pLKO.1 lentiviral vector (Addgene, USA), which was transfected to the hFOB1.19 cells via Lipofectamine 2000 protocol (Invitrogen, China). Control cells were transfected with non-targeting control shRNA (shCTRL, 5′-GCAAGCTGACCCTGAAGTTCAT-3′; Sigma Aldrich).

### Cell Counting Kit-8 (CCK-8) viability assay (*in vitro*)

The hFOB1.19 cells were initially plated onto 96-well plates at a density of 4,000 cells per well. The control group was left untreated. Following 24-h of treatment with or without Dex (200 μM) or with or without Dex (200 μM) + N7G treatment (20 to 200 μM), cell viability was assessed using a CCK-8 assay kit (Keygen, China). The absorbance values of CCK-8 were measured at 450 nm.

### Calcein acetoxymethyl-ester (AM) viability assay (*in vitro*)

The hFOB1.19 cells were initially seeded onto 96-well plates at a density of 4,000 cells per well. The control group remained untreated. Following 24-h of treatment with or without Dex (200 μM) or with or without Dex (200 μM) + N7G treatment (100 μM), 100 μL of 3 μM Calcein AM stock solution (Keygen) was added to each well and incubated for 30-min at 25°C. An Olympus IX71 inverted microscope (Japan) was used to capture images for assessing cell viability.

### Flow cytometry (*in vitro*)

Cell apoptosis of each group of hFOB1.19 human osteoblasts was measured by flow cytometry. Briefly, the hFOB1.19 human osteoblasts were harvested, washed with ice-cold PBS, and resuspended in the provided Annexin V binding buffer. Annexin V-FITC and PI were then added to the cell suspension and incubated in the dark for 15-min at room temperature. After staining, the samples were analyzed using the FACS Canto II flow cytometer (BD Biosciences, USA). A minimum of 10,000 events were recorded for each sample. The fluorescence signals for Annexin V-FITC (excitation/emission: 488/525 nm) and PI (excitation/emission: 488/620 nm) were detected in the appropriate channels. The data were analyzed using the BD FACSDiva software (version 8.0, BD Biosciences). Cells positive for Annexin V and/or PI were considered apoptotic, and the percentages of early apoptotic (Annexin V-positive, PI-negative) and late apoptotic/necrotic (Annexin V-positive, PI-positive) cells were quantified for each experimental group.

### TUNEL (terminal deoxynucleotidyl transferase dUTP nick end labeling) staining (*in vitro* and *in vivo*)

For the *in vitro* experiments, the hFOB1.19 cells were initially seeded onto 24-well tissue culture plates (1×10^4^ cells per well). Following different treatments, a TUNEL *In Situ* Cell Death Detection Kit (Roche, China) was used to quantitatively test cell apoptosis. Cells were co-stained with TUNEL and 4′,6-diamidino-2-phenylindole (DAPI) and visualized under a confocal fluorescent microscope (Leica, China). For each treatment, at least 600 cells in six random views (1×200 magnification) were included to calculate TUNEL ratio (% *vs* DAPI).

For the *in vivo* experiments, femoral head tissues collected from each group of rats were fixed in formaldehyde solution (pH=7.4) for 48-h and then decalcified in 10% ethylenediaminetetraacetic acid (EDTA) solution for 4-week. Then, decalcified bone tissue was embedded in paraffin and cut into 5‐μm‐thick sections for TUNEL staining (Roche) and subsequent morphological evaluation. Brownish‐yellow cells were identified as apoptotic cells.

### Western blot analysis (*in vitro* and *in vivo*)

In the *in vitro* experiments, total protein extracted from the hFOB1.19 cells was separated using radio-immunoprecipitation assay (RIPA) lysis buffer with proteinase inhibitor (Keygen). In the *in vivo* experiments, unilateral femoral heads of all rats were washed twice in ice-cold phosphate-buffered saline (PBS), dissolved in RIPA, and then ground in liquid nitrogen for about 30 min followed by 10-min microcentrifugation at 10,000 *g* for 10 min at 4°C. Protein concentrations were tested using the Bicinchoninic Acid Protein Assay Kit (Keygen). In total, 30 μg of protein from each sample was underwent sodium dodecyl-sulfate polyacrylamide gel electrophoresis. After separation, the proteins were transferred onto polyvinylidene difluoride (PVDF) membranes (Roche). The PVDF membranes were blocked for nearly 1 h and then incubated with the primary antibodies anti‐PKCζ (1:500; Abcam, UK), anti‐PKCζ phospho-T560 (1:500; Abcam), anti‐Bax (1:1000; Abcam), anti‐Bcl-2 (1:300; Abcam), anti‐cleaved caspase-3 (1:1000; Abcam), anti‐JNK (1:1000; Abcam), anti‐JNK phospho-T183 (1:2000; Abcam), anti‐c-jun (1:1000; Abcam), anti‐c-jun phospho-S63 (1:2000; Abcam), and anti‐tubulin (1:5000; Abcam) at 4°C for about 24 h. This was followed by incubation with the corresponding secondary antibodies (1:2000; Abcam) at room temperature for nearly 1 h. Protein bands were visualized using BeyoECL Moon (Keygen).

### Hematoxylin and eosin (H&E) staining (*in vivo*)

Femoral head tissues collected from each group of rats were fixed in formaldehyde solution (pH=7.4) for 48 h and then decalcified in 10% EDTA solution for 4 weeks. Decalcified bone tissue was then embedded in paraffin and cut into 5‐μm‐thick sections for H&E staining and subsequent morphological evaluation. Under a BX61 microscope (Olympus), empty bone lacunae of bone cells were observed. Histology was assessed by two independent observers who were blinded to the dietary condition and the percentage of empty lacunae was recorded.

### Micro-computed tomography (micro-CT) scanning (*in vivo*)

The femoral head tissues collected from each group of rats were subjected to micro-CT scanning before and after decalcification in a 10% EDTA solution for 4 weeks. Scans were performed at a resolution of 9 μm per pixel. Trabecular bone was separated from the bone marrow for analysis, which included trabecular thickness (Tb.Th), trabecular separation (Tb.Sp), bone volume per tissue volume (BV/TV), and trabecular number (Tb.N). The analyses were focused on the femoral head, with measurements conducted approximately 1 mm from the epiphyseal growth plate (EGP) and 0.5 mm below the articular cartilage. Three planes (coronal, sagittal, and transverse sections) of representative samples from each group were generated using DataViewer software (Bruker Micro-CT, USA).

### Statistical analysis

Statistical analysis was performed using GraphPad Prism 8.0 software (USA). Data are reported as means±SD. Data normality was determined by using Shapiro-Wilk test. Statistical comparisons were made by one-way analysis of variance (ANOVA) with Tukey's range test. An adjusted P<0.05 was considered significant after Tukey's corrections for multiple comparisons.

## Results

### N7G attenuated Dex-induced osteoblasts apoptosis via inhibiting PKC**ζ** activation

**Figure 1:**
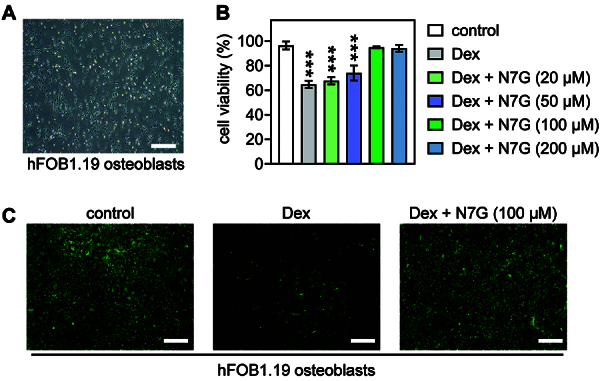
Naringenin-7-O-β-D-glucuronide (N7G) reversed the reduction of cell viability of osteoblasts induced by dexamethasone (Dex). **A**, Microscopic images of human hFOB1.19 osteoblasts (scale bar 500 μm). **B**, Viability of osteoblasts after 24 h of Dex stimulation (200 μM), with or without N7G treatment (20-200 μM). Data are reported as means±SD. ***P<0.001 compared with control (no treatment) group (ANOVA). **C**, Viability of osteoblasts after 24 h of treatment with or without Dex (200 μM) and with Dex (200 μM) + N7G treatment (100 μM), as assessed by calcein-acetoxymethyl-ester staining (scale bar 500 μm).

Figure 1A shows the morphology of hFOB1.19 cells. We found that cell viability suppressed by Dex stimulation (200 μM, (4)) was significantly rescued by 100 μM N7G after 24 h (Figure 1B and C). Thus, 100 μM N7G was used in subsequent experiments.

**Figure 2:**
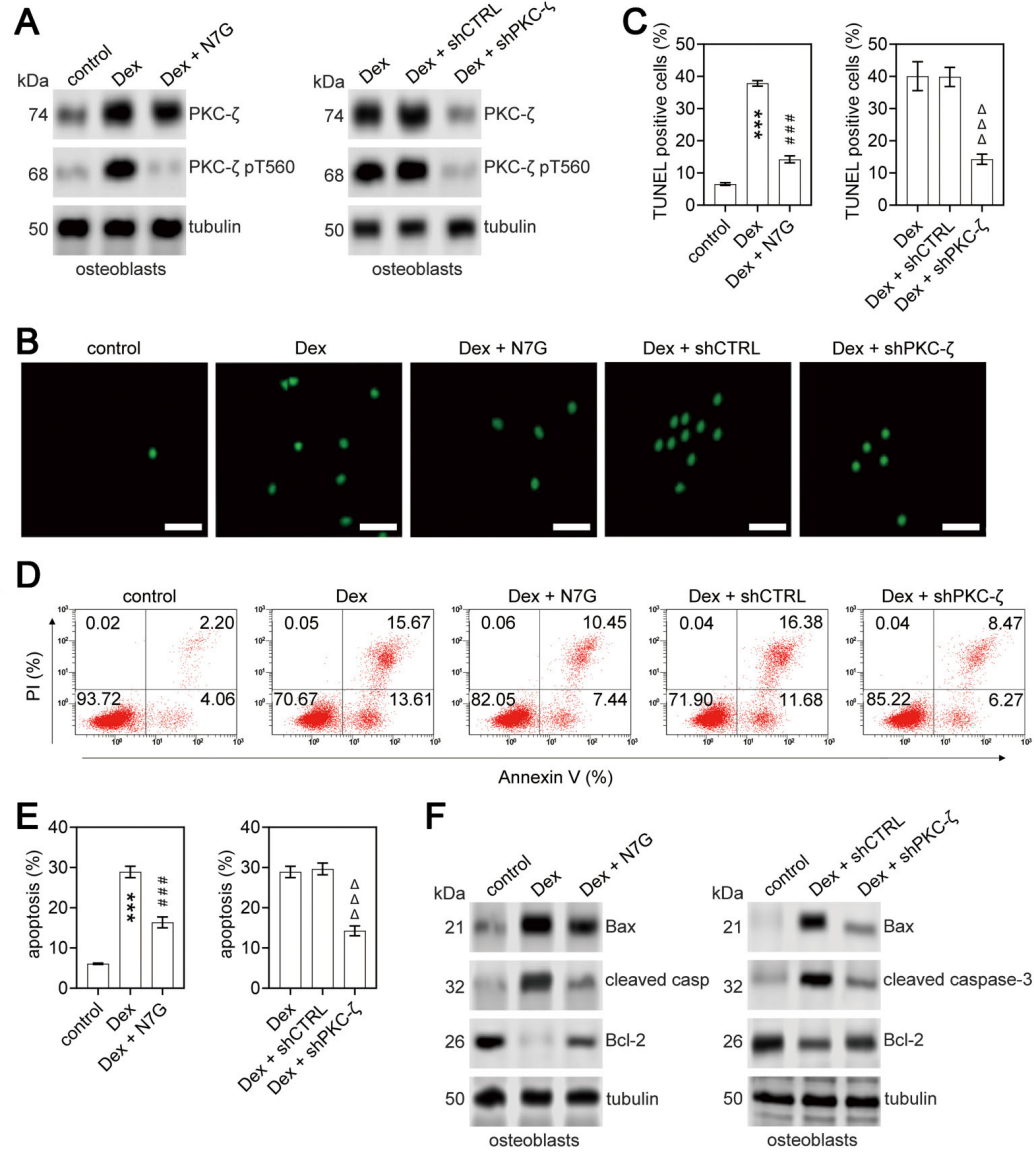
Effects of PKCζ on apoptosis of dexamethasone (Dex)-stimulated osteoblasts. **A**, Western blot analysis was performed to assess the protein expression levels of PKCζ and its T560 phosphorylation (PKCζ pT560) in osteoblasts from different experimental groups: control (no treatment), Dex or Dex + naringenin-7-O-β-D-glucuronide (N7G) (left panel), and Dex, Dex + shCTRL, or Dex + shPKCζ (right panel). Tubulin was used as an internal control. **B** and **C**, Images of TUNEL staining (B) in osteoblasts from different experimental groups (scale bar 200 μm). Quantitation of TUNEL-positive osteoblasts (C). Data are reported as means±SD. ***P<0.001 *vs* control group and *vs* Dex + N7G group; ^###^P<0.001 *vs* control group; ^ΔΔΔ^P<0.001 *vs* Dex group and *vs* Dex + shCTRL group (ANOVA). **D** and **E**, Annexin V/PI double staining (**D**) in osteoblasts from different experimental groups. **E**, Quantitation of apoptosis in osteoblasts. Data are reported as means±SD. ***P<0.001 *vs* control group and *vs* Dex + N7G group; ^###^P<0.001 *vs* control group; ^ΔΔΔ^P<0.001 *vs* Dex group and *vs* Dex + shCTRL group (ANOVA). **F**, Western blot analysis of the protein expression levels of Bax, cleaved caspase-3, and Bcl-2 in osteoblasts from different experimental groups. Tubulin was used as an internal control.

Dex stimulation increased PKCζ protein expression and activation (measured by PKCζ T560 phosphorylation), and N7G treatment reversed this (Figure 2A). TUNEL staining and Annexin V/PI double staining (for cell apoptosis) also showed that N7G treatment reversed the apoptosis rate of osteoblasts following Dex stimulation (Figure 2B-E). In addition, the expression of pro-apoptotic proteins Bax and cleaved caspase-3 was significantly upregulated, whereas the expression of anti-apoptotic Bcl-2 was downregulated in Dex-stimulated osteoblasts (Figure 2F). As expected, Bcl-2 expression increased while Bax and cleaved caspase-3 decreased in Dex-stimulated osteoblasts following N7G treatment (Figure 2F). Similar cellular protection was observed in Dex-stimulated osteoblasts following PKCζ knockdown (Figures 2A-F), consistent with previous reports. Thus, N7G treatment inhibited PKCζ activation, thereby reversing the cytotoxic effects in the Dex-induced ONFH cellular model.

### N7G decreased GC-induced ONFH and osteoblasts apoptosis in rats

**Figure 3:**
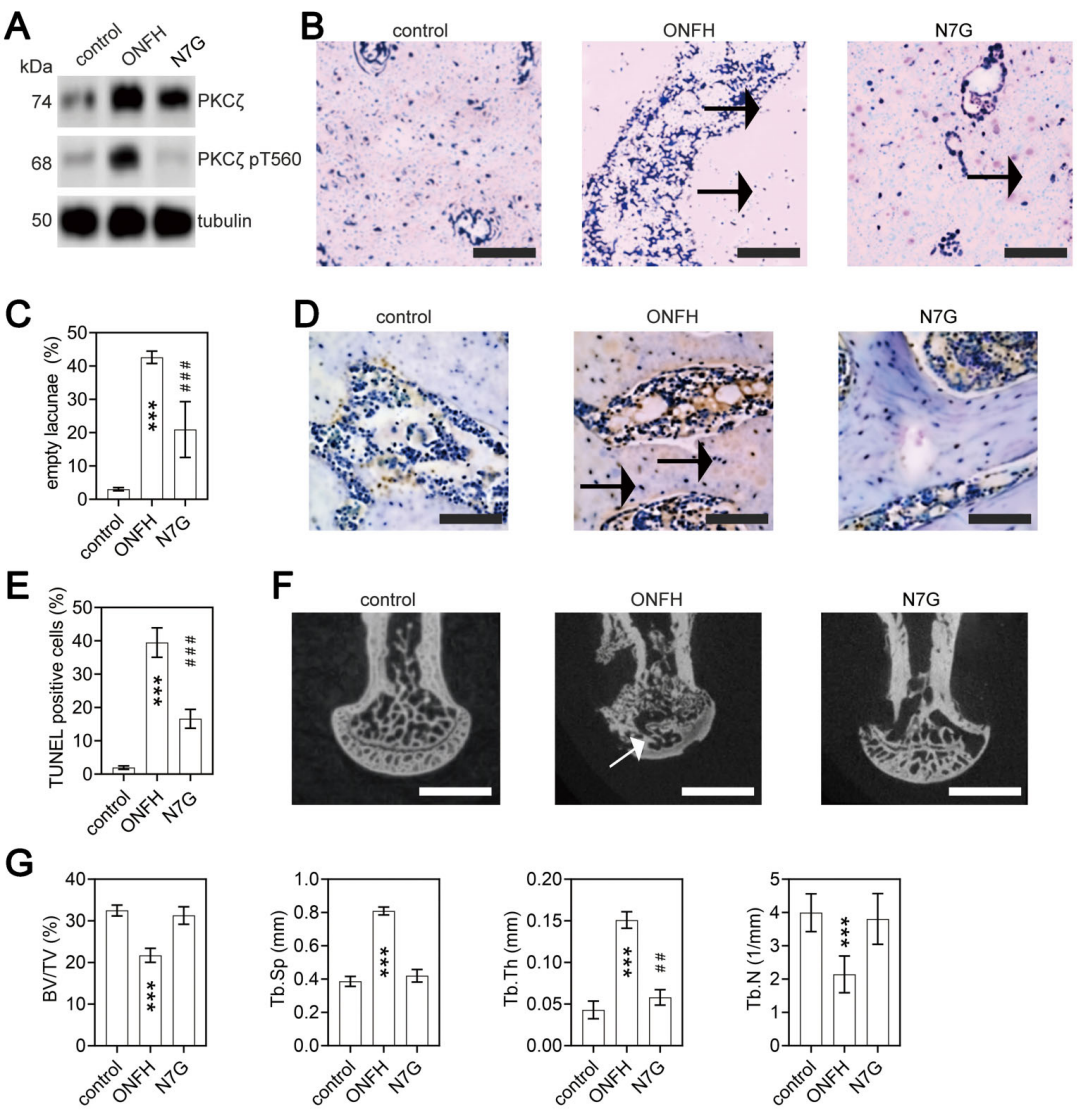
Evaluation of osteonecrosis and apoptosis in naringenin-7-O-β-D-glucuronide (N7G)‐treated osteonecrosis of the femoral head (ONFH) rats. Control: untreated rats; ONFH: rats treated with dexamethasone (Dex); N7G: rats treated with Dex and with the PKCζ inhibitor N7G. **A**, Western blot analysis was performed to assess the protein expression levels of PKCζ and its T560 phosphorylation (PKCζ pT560) in the unilateral femoral heads from different experimental rat groups. Tubulin was used as an internal control. **B** and **C**, H&E staining (scale bar: 100 μm) of femoral heads of rats in the control, ONFH, and N7G groups. Arrows indicate empty lacunae (**B**). Quantitative analysis of the empty lacunae rate in the three groups (**C**). Data are reported as means±SD. **D** and **E**, TUNEL staining (scale bar: 100 μm) of femoral heads of rats in the groups (arrows indicate TUNEL‐positive cells) and quantitative analysis of the proportion of TUNEL-positive cells in the three groups. Data are reported as means±SD. **F** and **G**, Micro-CT analysis of femoral heads of rats in groups (arrows indicate regions of decreased trabecular density; scale bar 1 mm) and quantitative analysis of bone volume per tissue volume (BV/TV), trabecular separation (Tb.Sp), trabecular thickness (Tb.Th), and trabecular number (Tb.N) in the three groups. Data are reported as means±SD. ***P<0.001 *vs* control group and *vs* N7G group. ^##^P<0.01; ^###^P<0.001 *vs* ONFH group (ANOVA).

We conducted animal experiments to further verify the effects of N7G on femoral head necrosis induced by GC in rats. Similar to the Dex-induced ONFH cellular model, the expression levels of PKCζ protein and its T560 phosphorylation were increased in the ONFH rat bone tissues. In addition, N7G treatment attenuated all these effects (Figure 3A). HE staining of femoral head necrosis (Figure 3B) in the ONFH group was characterized by the disordered arrangement of the trabecular bone and a large number of empty bone lacunae. In contrast, the trabecular shape appeared better preserved in the N7G group compared to the ONFH group. Empty bone lacunae were present in N7G but were fewer than those in ONFH group. The control, ONFH, and N7G groups had empty bone lacunae rates of 2.3, 39.1, and 19.2%, respectively (Figure 3C).

We also used TUNEL staining to detect apoptosis in rat femoral head cells (Figure 3D). The rate of TUNEL-positive cells was 28.3% in the ONFH model group and 11.3% in N7G. Thus, the proportion of apoptotic cells to normal cells was significantly lower in N7G than in ONFH group (Figure 3E). These results further showed the inhibitory effect of N7G on femoral head necrosis and osteoblasts apoptosis.

Furthermore, micro-CT scanning and reconstruction analysis showed the trabeculae in the control group were clearly visible, tightly connected, and the surface of the femoral head was smooth without collapse. In contrast, the ONFH group exhibited bones with sparse trabeculae with greater distance between trabecular structures. Conversely, the N7G group had trabecular bones with smaller spacing compared to the ONFH group (Figure 3F and G; Table 1). Overall, these results suggested that N7G treatment significantly protected against microstructural deterioration of the rat femoral head, enhancing and standardizing the quantity and shape of bone trabeculae, although there was a statistical difference in the Tb.Th results between the N7G group and the control group in the reconstruction analysis derived from micro-CT scanning (Figure 3G; Table 1).

**Table 1:** Comparison of femoral head microarchitecture parameters in control, osteonecrosis of the femoral head (ONFH), and naringenin-7-O-β-D-glucuronide (N7G) group rats.

Variables	Control	ONFH	N7G
BS/BV (%)	32.49±1.31	21.73±1.67***	31.29±2.09
Tb.Sp (mm)	0.39±0.03	0.81±0.02***	0.42±0.04
Tb.Th (mm)	0.04±0.01	0.15±0.01***	0.06±0.01^##^
Tb.N (/mm)	3.99±0.57	2.14±0.55***	3.81±0.76

Control: untreated rats; ONFH: rats treated with dexamethasone (Dex); N7G: rats treated with Dex and with the PKCζ inhibitor N7G. Data are reported as means±SD. ***P<0.001 *vs* control group and *vs* N7G group. ^##^P<0.01 *vs* ONFH group (ANOVA). BS/BV: bone volume per tissue volume; Tb.Sp: trabecular separation; Tb.Th: trabecular thickness; Tb.N: trabecular number.

### PKC**ζ** activated JNK/c-Jun signaling involved in GC-induced ONFH

**Figure 4:**
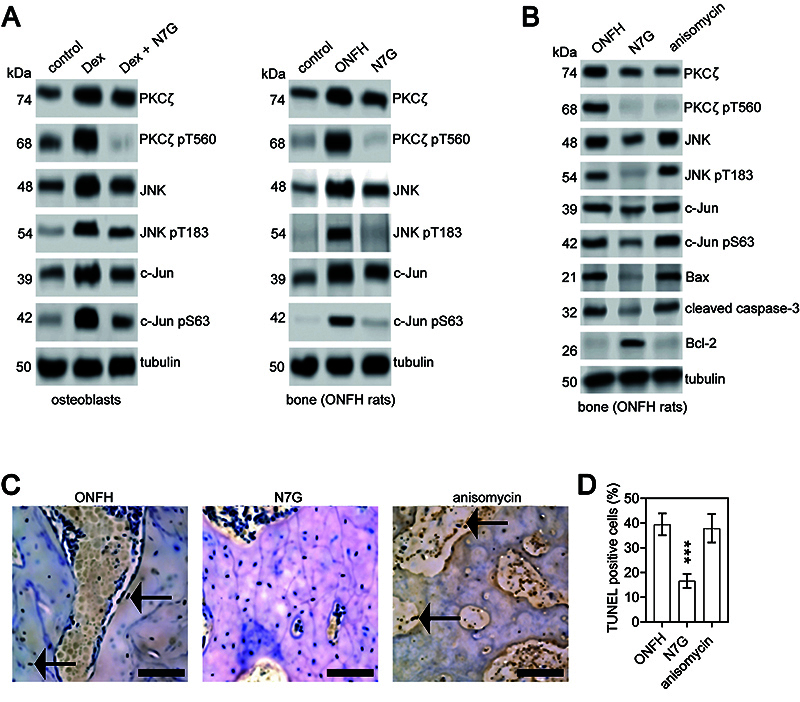
Anisomycin reverses the benefits of naringenin-7-O-β-D-glucuronide (N7G) on osteoblast apoptosis in osteonecrosis of the femoral head (ONFH) rats. ONFH: rats treated with dexamethasone (Dex); N7G: rats treated with Dex with the PKCζ inhibitor N7G; anisomycin: rats treated with Dex with the PKCζ inhibitor N7G and the JNK agonist anisomycin. **A**, Western blot analysis was performed to assess the protein expression levels of PKCζ and its T560 phosphorylation (PKCζ pT560), JNK and its T183 phosphorylation (JNK pT183), and c-Jun and its S63 phosphorylation (c-Jun pS63) in osteoblasts (left panel) and in the unilateral femoral heads from the experimental rat groups (right panel). Tubulin was used as an internal control. **B**, Western blot analysis of the protein expression levels in the unilateral femoral heads from the experimental groups. Tubulin was used as an internal control. **C** and **D**, TUNEL staining (scale bar: 100 μm) of femoral heads of rats (arrows indicate TUNEL‐positive cells) and quantitative analysis of the proportion of TUNEL-positive cells in the three groups. Data are reported as means±SD. ***P<0.001 *vs* ONFH group and *vs* anisomycin group (ANOVA).

**Figure 5:**
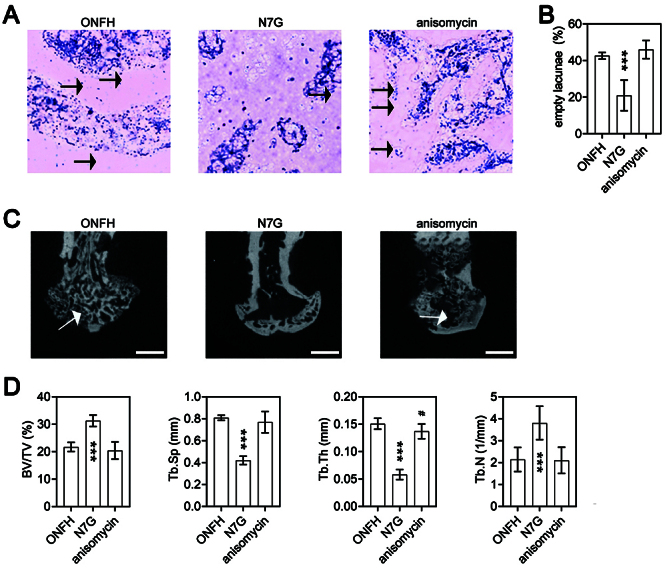
Anisomycin counteracts naringenin-7-O-β-D-glucuronide (N7G) mediated bone microstructure protection in osteonecrosis of the femoral head (ONFH) rats. ONFH: rats treated with dexamethasone (Dex); N7G: rats treated with Dex with the PKCζ inhibitor N7G; anisomycin: rats treated with Dex with the PKCζ inhibitor N7G and the JNK agonist anisomycin. **A** and **B**, H&E staining (scale bar: 100 μm) of femoral heads of rats (arrows indicate empty lacunae) and quantitative analysis of the empty lacunae rate in the three groups. Data are reported as means±SD. **C** and **D**, Micro-CT analysis of femoral heads of rats (arrows indicate regions of decreased trabecular density; scale bar 1 mm) and quantitative analysis of bone volume per tissue volume (BV/TV), trabecular separation (Tb.Sp), trabecular thickness (Tb.Th), and trabecular number (Tb.N) in the three groups. Data are reported as means±SD. ***P<0.001 *vs* ONFH group and *vs* anisomycin group. ^#^P<0.05 *vs* ONFH group (ANOVA).

JNK and its downstream transcription factor c-Jun have been identified as crucial mediators of osteoblast apoptosis in the development and progression of ONFH. Previous research has shown that PKCζ can activate JNK (21,22). Our findings demonstrated that treatment with N7G not only reduced PKCζ activation, but also inhibited JNK/c-Jun signaling in both a Dex-induced ONFH cellular model and GC-induced ONFH rats (Figure 4A). To investigate whether the downregulation of JNK/c-Jun signaling was responsible for the inhibition of PKCζ following N7G treatment, anisomycin was used to activate JNK in GC-induced ONFH rats treated with N7G. We found that anisomycin treatment did not affect N7G-induced reduction of PKCζ activation in ONFH rats, but it did rescue the activation of JNK/c-Jun signaling in the bone tissues of N7G-treated ONFH group (Figure 4B). In addition, compared with the N7G group, the expression of pro-apoptotic proteins Bax and cleaved caspase-3 was significantly upregulated, while the expression of anti-apoptotic Bcl-2 was downregulated in N7G-treated ONFH rats after anisomycin treatment (Figure 4B). Furthermore, TUNEL (Figure 4C and D) and H&E staining (Figure 5A and B) revealed that anisomycin treatment increased the proportion of apoptotic cells to normal cells (Figure 4D) and the percentage of empty lacunae (Figure 5B) compared to the N7G group in ONFH rats. Moreover, compared to N7G group, the subchondral trabeculae in the anisomycin group were reversed (Figure 5B). The quantitative analysis of BV/TV and Tb.N, as detected by micro-CT, revealed significantly lower values in the anisomycin group compared to the N7G group. Conversely, Tb.Sp and Tb.Th were significantly higher in the anisomycin group compared to the N7G group (Figure 5C and D; Table 2), with the anisomycin group showing slightly lower Tb.Th results than the ONFH group (Figure 5D; Table 2). Therefore, these data indicated that the activation of JNK/c-Jun signaling was required for PKCζ-induced osteoblast injury in the progression of ONFH.

**Table 2:** Comparison of femoral head microarchitecture parameters in osteonecrosis of the femoral head (ONFH), naringenin-7-O-β-D-glucuronide (N7G), and anisomycin group rats.

Variables	ONFH	N7G	Anisomycin
BS/BV (%)	21.73±1.67	31.29±2.09***	20.43±3.12
Tb.Sp (mm)	0.81±0.02	0.42±0.04***	0.77±0.1
Tb.Th (mm)	0.15±0.01	0.06±0.01***	0.14±0.01^#^
Tb.N (1/mm)	2.14±0.55	3.81±0.76***	2.1±0.6

ONFH: rats treated with dexamethasone (Dex); N7G: rats treated with Dex and with the PKCζ inhibitor N7G; anisomycin, rats treated with Dex and the PKCζ inhibitor N7G and the JNK agonist anisomycin. Data are reported as means±SD. ***P<0.001 *vs* ONFH group and *vs* anisomycin group. ^#^P<0.05 *vs* ONFH group (ANOVA). BS/BV: bone volume per tissue volume; Tb.Sp: trabecular separation; Tb.Th: trabecular thickness; Tb.N: trabecular number.

## Discussion

ONFH is a debilitating joint disease characterized by progressive bone destruction, leading to femoral head collapse and severe hip dysfunction (1,2). While GC use is a major risk factor, the precise molecular mechanisms underlying ONFH remain elusive (1,2). Recent studies have implicated aberrant activation of PKCζ in the pathogenesis of ONFH, particularly in GC-induced osteoblast apoptosis (19).

PKCζ, an atypical member of the PKC family, is uniquely activated through protein-protein interactions rather than by diacylglycerol or phorbol esters (23,24). In the context of ONFH, PKCζ activation has been linked to excessive GC usage, which induces oxidative stress and triggers a cascade of events leading to osteoblast apoptosis (19). This connection between PKCζ and ONFH pathogenesis has opened new avenues for therapeutic intervention.

Naringenin, a flavonoid abundant in citrus fruits, has demonstrated promising anti-apoptotic effects in various disease models (13-15). Importantly, it has also been identified as a natural inhibitor of PKCζ (13). Building on these findings, our study focused on N7G, a metabolite of naringenin with enhanced bioavailability (14,15). We hypothesized that N7G could mitigate ONFH progression by targeting the PKCζ signaling pathway. Our investigations revealed that N7G effectively attenuates GC-induced osteoblast apoptosis and alleviates ONFH in both cellular and rat models. We observed that GC treatment increased PKCζ protein expression and activation (measured by T560 phosphorylation), while N7G treatment reversed these effects. This PKCζ inhibition coincided with reduced osteoblast apoptosis and improved bone microstructure in ONFH rats, consistent with previous studies implicating PKCζ in ONFH development (19).

Furthermore, we found that N7G treatment inhibited the JNK/c-Jun signaling pathway, a known mediator of osteoblast apoptosis in ONFH (25-30). Interestingly, while N7G reduced both PKCζ and JNK/c-Jun activation, selective JNK activation using anisomycin (20) in N7G-treated ONFH rats rescued JNK/c-Jun signaling without affecting PKCζ inhibition. This suggested that JNK/c-Jun signaling may be downstream of PKCζ in the ONFH context, providing new insights into the signaling cascades involved in GC-induced osteoblast apoptosis.

However, it is crucial to acknowledge the limitations of our study. While our results demonstrated a protective effect of N7G on ONFH pathogenesis, we must exercise caution in attributing this effect solely to PKCζ inhibition. As an antioxidant compound, N7G likely has broad effects on cellular redox status, potentially influencing multiple signaling pathways simultaneously.

The protective effects we observed may result from a combination of direct and indirect mechanisms. N7G's antioxidant properties could lead to a general reduction in ROS, which play a crucial role in osteoblast apoptosis and ONFH development (31-33). This broad antioxidant effect could downregulate several ROS-sensitive signaling pathways, including but not limited to PKCζ and JNK/c-Jun. Importantly, PKCζ itself plays a key role in ROS regulation, acting as both a target and a mediator of oxidative stress. The interplay between N7G's antioxidant effects and its potential direct inhibition of PKCζ adds complexity to the interpretation of our results.

Furthermore, our study did not conclusively demonstrate that N7G selectively targeted PKCζ in the context of ONFH. Additional experiments, such as *in vitro* kinase assays or the use of more specific PKCζ inhibitors, would be necessary to establish a direct interaction between N7G and PKCζ in this pathological context.

## Conclusion

In conclusion, while our study provided promising evidence for the protective effects of N7G in GC-induced ONFH, further research is needed to fully elucidate the underlying mechanisms. The potential of N7G as a therapeutic agent for ONFH remains an exciting avenue for future investigation. However, a careful consideration of its complex actions as both an antioxidant and a potential signaling modulator is necessary for a comprehensive understanding of its therapeutic potential. Future studies should focus on disentangling the direct and indirect effects of N7G on PKCζ and other relevant signaling pathways in ONFH pathogenesis.
